# Interfacial Electronic Coupling in Si@SiC@EG Core–Shell Architectures Enables High-Capacity and Long-Life Lithium-Ion Batteries

**DOI:** 10.3390/molecules30234517

**Published:** 2025-11-22

**Authors:** Huangyu Zhao, Sihao He, Changlong Sun, Kesheng Gao, Honglin Li, Qiuju Zheng, Lingshan Geng, Yan-Jie Wang, Enyue Zhao, Yuanyuan Zhu

**Affiliations:** 1 New Energy and Advanced Functional Materials Group, School of Materials Science and Engineering, Dongguan University of Technology, Dongguan 523808, China; huangyuzhao12@163.com (H.Z.); sihaohe0808@163.com (S.H.); 2 Songshan Lake Materials Laboratory, Dongguan 523808, China; gaokesheng98@gmail.com; 3 Key Laboratory of Spin Electron and Nanomaterials of Anhui Higher Education Institutes, Suzhou University, Suzhou 234000, China; changlongsun@qust.edu.cn (C.S.); 15555942181@163.com (L.G.); 4 College of Materials Science and Engineering, Qingdao University of Science and Technology, Qingdao 266042, China; 13176573302@163.com; 5 School of Materials Science and Engineering, Qilu University of Technology, Jinan 250353, China; qlzhengqj@163.com

**Keywords:** silicon anode, core–shell structure, lithium-ion battery, interfacial engineering

## Abstract

Silicon anodes have attracted considerable attention as next-generation lithium-ion battery materials owing to their exceptionally high theoretical capacity. However, their practical application remains limited by severe volume fluctuations during cycling, which lead to rapid capacity fading. In this work, a Si@SiC@ epitaxial Graphene (EG) core–shell nanocomposite is constructed through in situ epitaxial growth to overcome these challenges. The SiC interlayer functions as a robust mechanical buffer, accommodating the volume expansion of silicon during lithiation and delithiation, while the external graphene shell offers high electronic conductivity, structural resilience, and may provide additional Li^+^ storage sites. Structural and electrochemical characterizations, including ex situ X-ray diffraction, in situ Raman spectroscopy, and ex situ X-ray photoelectron spectroscopy, verify the reversible Li^+^ insertion/extraction and the preservation of structural integrity without phase collapse. The Si@SiC@EG anode delivers a high reversible capacity of 1747 mAh g^−1^ at 0.1 A g^−1^, outstanding rate performance, and remarkable durability, maintaining 872 mAh g^−1^ after 2000 cycles at 1 A g^−1^. Density functional theory calculations further indicate that strong interfacial coupling effectively lowers Li^+^ migration barriers, thereby improving ion transport kinetics. These findings highlight the potential of the Si@SiC@EG heterostructure as a viable platform for high-energy-density lithium-ion storage.

## 1. Introduction

Electrochemical energy storage devices are considered the most practical approach for storing renewable energy generated by solar and wind power [1]. Lithium-ion batteries (LIBs) benefit from their excellent energy density, non-memory characteristics, and low self-discharge performance, standing out among a wide range of novel storage devices [2,3,4]. However, the conventional carbon-based anodes are approaching the theoretical capacity limit, and the exploration of alternative anode materials has become a focal point in recent years. Therefore, silicon-based nanocrystal materials with high energy density are considered as feasible alternative materials [5,6,7]. The alloying reaction of silicon with lithium demonstrates an ultra-high theoretical capacity (4200 mAh g^−1^) and relatively low operation voltage (~0.1 V, vs. Li/Li^+^) [8,9]. However, the electrochemical performance of the silicon anode is still restricted by low conductivity, poor ion diffusion, and huge volume expansion during the alloying/dealloying process. To overcome these issues, various strategies have been proposed, including pre-lithiation, structural design (such as nanotubes, nanowires, and thin films), and component modulation (e.g., Ga-Si, Zn-Si, Cu-Si, and Ge-Si) [10,11]. However, the regeneration of the solid-electrolyte interphase (SEI) film during lithiation can reduce coulomb efficiency (CE), convert recyclable lithium-ion into “dead lithium”, and even spark multiple side reactions with the electrolyte.

Therefore, numerous scholars have attempted to address the challenges by exploring advanced core–shell structures in achieving idealized Si-based anodes. The core–shell structure is a proven and versatile strategy for enhancing the structural stability of silicon-based anodes. The shell acts as a protective layer that buffers the large volume expansion and contraction of silicon during lithiation and delithiation [12,13]. Therefore, it can reduce the mechanical stress and preserve the structural integrity of the Si-based anodes. In addition, this artificially engineered architecture also creates continuous pathways for electron and ion transport, which improves reaction kinetics and maintains a stable anode-electrolyte interface [14]. Depending on the selected shell materials, it can generate entirely unanticipated effects, such as limited side reactions and enhanced electrical conductivity. Notably, the durability improvement often comes at the expense of capacity, and the conductivity enhancement tends to result in considerable reduction in volumetric energy density [15]. Furthermore, the complex fabrication process and high costs associated with these advanced structures pose a significant hindrance to the widespread application of Si-based anodes. The integration of conductive and flexible outer layer through a controlled in situ growth process is considered to be one of the most effective strategies to solve these issues. More importantly, the shell thickness and composition of the core–shell Si-based anode can be precisely controlled through this in situ growth strategy. This can further strengthen the interfacial interaction between the Si core and the shell, while ensuring efficient electron and ion transport [16]. By optimizing the core–shell structure through an in situ growth strategy, it is possible to alleviate the volume expansion and enhance the energy density without sacrificing capacity or cycling stability. Among different shell materials, silicon carbide has drawn attention because of its high mechanical strength and chemical stability [17]. A SiC layer can firmly connect with the silicon core, which improves the bonding at the interface and prevents detachment during long-term cycling. At the same time, the rigid and stable nature of SiC provides strong support that helps buffer the stress caused by repeated volume changes of silicon. This protection greatly slows down the structural damage of the active material and improves cycling stability. In addition, carbon-based layers such as epitaxial graphene (EG) offer complementary advantages. Their high electrical conductivity creates efficient ways for electrons, which reduces internal resistance and enhances reaction kinetics [18]. Moreover, the flexible and layered structure of graphene can adapt to the expansion and contraction of the core, maintaining close contact and reducing interfacial stress. By combining a robust carbide interlayer with a conductive and flexible carbon shell, it is possible to balance mechanical stability with high electronic conductivity, leading to improved overall battery performance [19]. Compared with other widely studied strategies, such as SiO_x_ coatings, carbon encapsulation, hollow/yolk–shell designs, or alloying, the combination of a robust SiC interlayer and a flexible, conductive epitaxial graphene shell in the present design offers strong interfacial bonding, efficient electron and ion transport, and superior cycling stability, highlighting its competitive performance and practical potential [20].

This work reports a practical synthesis strategy to produce double-shell Si@SiC@EG nanocomposites, where a silicon carbide layer is epitaxially grown in situ on the silicon core. The double-shell structure effectively mitigates the volume expansion and guarantees the cycling durability of the Si@SiC@EG anode. The engineered interfaces enhance coupling between the core and shells, which not only provide continuous diffusion channels to lower the ion transport barrier but also induce charge re-distribution that generates built-in electric fields for faster ion movement. In this work, the combination of an active Si core, a SiC interlayer, and an in situ epitaxially grown graphene shell further strengthens interfacial bonding and electronic coupling while facilitating directional Li ions transport, thereby mitigating volume expansion and stabilizing the SEI layer. The formation of stable Si-C bonds effectively anchors the silicon core within the carbide framework, which alleviates structural collapse during repeated expansion and contraction. Benefiting from this enhanced stability, the anode can maintain a reversible capacity of 872 mAh g^−1^ after 2000 cycles at a current density of 1 A g^−1^. Ex situ XRD and XPS analyses, together with in situ Raman spectroscopy, confirm that the lithium-storage process involves a combined intercalation-alloying mechanism, in which SiC and EG mainly undergo intercalation/pseudocapacitive reactions, while the Si core participates in reversible alloying with Li^+^. This finding suggests that the anode undergoes significant reversible structural changes during cycling, thereby ensuring its long-term electrochemical stability and extending its service life. Therefore, the Si@SiC@EG double-shell design provides a practical and scalable way to overcome the long-standing challenges of silicon anodes, offering strong potential for integration into next-generation high-performance lithium-ion batteries.

## 2. Results and Discussion

**Figure 1:**
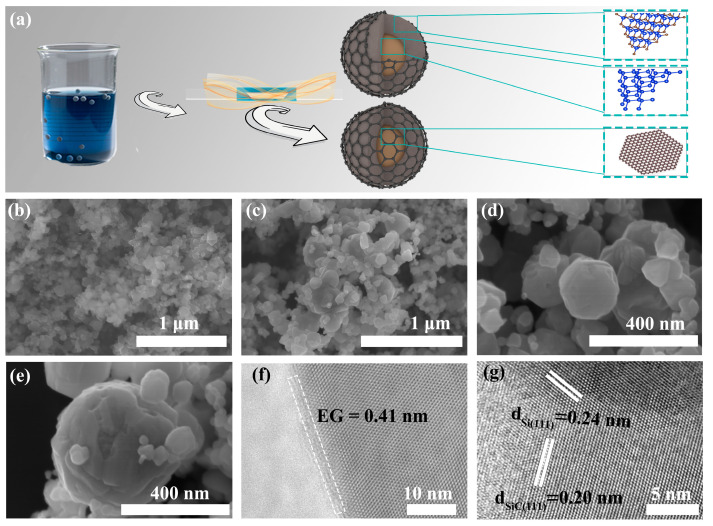
(**a**) Schematic of the synthesis of Si@SiC@EG. (**b**–**e**) Different magnification SEM of Si@SiC@EG. (**f**,**g**) HRTEM images of Si@SiC@EG.

Figure 1a shows a schematic of the synthesis of Si@SiC@EG, in which the Si@SiC@EG nanoparticles (NPs) are prepared by in situ epitaxial growth. Specific experimental parameters are detailed in the Supporting Information S1. The scanning electron microscopy (SEM) results for Si@SiC@EG are presented in Figure 1b–e. During high-temperature calcination, the small NPs spontaneously reduce surface energy, thus assuming a regular spherical morphology. To further enhance the stability, small NPs are agglomerated together to form Si@SiC@EG NPs with a thermodynamically stable state, and the formation of an external carbon layer ultimately results in an enlarged particle size. The structural characteristics of the Si@SiC@EG nanocomposite are further elucidated by high-resolution transmission electron microscopy (HRTEM), as shown in Figure 1f,g. A distinct and continuous outer boundary corresponding to the epitaxial graphene (EG) shell is clearly visible in Figure 1f, with a measured interlayer spacing of approximately 0.41 nm, confirming the successful formation of a thin, uniform EG coating on the Si@SiC surface. This EG layer not only may provide abundant lithium-ion storage sites but also promotes fast Li^+^ transport through the interfacial built-in electric field, thereby improving the reaction kinetics during cycling. To further expose the inner core–shell structure, the Si@SiC@EG NPs are subjected to ultrasonic fragmentation, as illustrated in Figure 1g. After the removal of the external EG shell, a clear heterointerface between Si and SiC can be observed [21]. The upper region, characterized by a darker contrast, exhibits a lattice spacing of 0.24 nm, corresponding to the (111) plane of crystalline Si, while the lower region with a lighter contrast displays a lattice spacing of 0.20 nm, corresponding to the (111) plane of SiC, as further confirmed by the FFT patterns shown in Figure S1. The well-defined interface and distinct lattice fringes provide clear evidence of the high crystallinity of both phases and confirm the successful in situ epitaxial growth of SiC on the Si core. This rationally engineered Si@SiC@EG architecture can effectively buffer the large volume changes during lithiation/delithiation and enhance the structural integrity and cycling stability of the anode [22].

**Figure 2:**
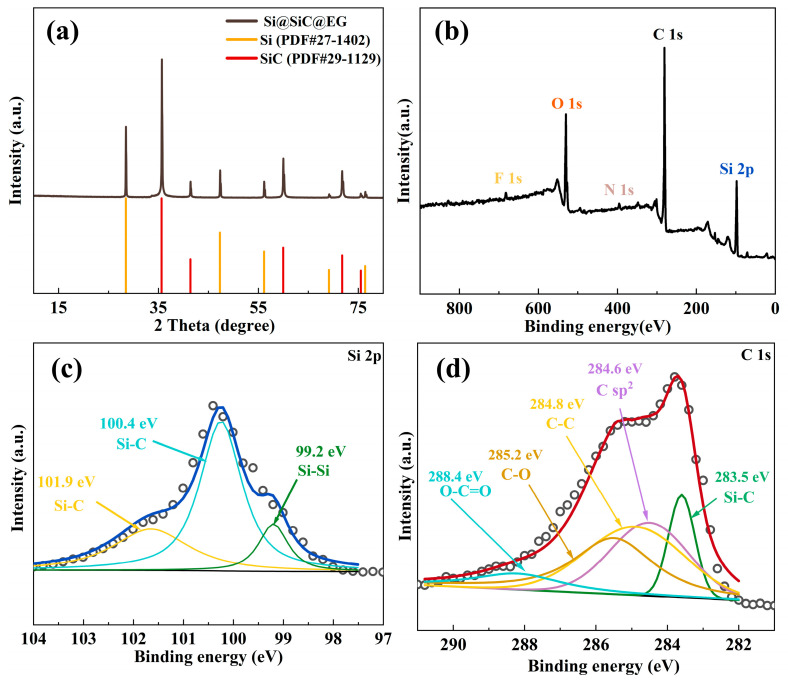
(**a**) XRD pattern, (**b**) XPS survey spectra, (**c**) Si 2p spectra, (**d**) C 1s spectra of Si@SiC@EG.

The crystal structure of Si@SiC@EG is investigated by X-ray powder diffraction (XRD), as shown in Figure 2a. The distinct diffraction peaks located at 35.6, 41.4, and 60.1° can correspond to the (111), (200), and (220) crystal planes of SiC (PDF#29-1129), respectively [23,24,25]. And the diffraction peaks at 28.4, 47.3, and 56.1° can be indexed to (111), (220), and (311) crystal planes of Si (PDF#27-1402) [26]. To unambiguously confirm the presence of EG, an enlarged XRD spectrum is presented as the inset in Figure S2. Within this region (20–25°), distinguishable peaks exhibited significantly different characteristics from pure-phase C, thereby conclusively establishing the presence of EG [27]. The presence of EG not only substantially enhances the electrical conductivity but also effectively eliminates charge accumulation, thereby significantly facilitating the electrochemical kinetics. The elemental composition and bonding states of the Si@SiC@EG heterostructure are examined using X-ray photoelectron spectroscopy (XPS). The survey spectrum (Figure 2b) reveals the presence of Si, C, and O elements, confirming the basic chemical composition of the composite [28]. As shown in Figure 2c, the Si 2p spectrum can be fitted into three components corresponding to Si-Si (99.2 eV) and Si-C bonds (100.4 and 101.9 eV) [29,30]. The appearance of the Si-Si peak not only demonstrates the preservation of the silicon core but also suggests partial surface decomposition of SiC during thermal processing. The C 1s spectrum (Figure 2d) further elucidates the interfacial structure. It can be fitted with three peaks at 283.5, 284.6, and 285.7 eV, assigned to Si-C bonds, sp^2^-hybridized carbon, and sp^3^-hybridized carbon, respectively [31]. The Si-C feature at 283.5 eV verifies the formation of a SiC phase at the interface [32]. The pronounced sp^2^ peak at 284.6 eV originates from epitaxial graphene layers generated through high-temperature decomposition of the SiC surface, confirming the successful formation of a conductive carbon shell. Meanwhile, the C-C bonds at 284.8 eV reflects the presence of a buffer layer between the SiC interlayer and the outer graphene coating [32]. The C-O, O-C=O mainly originate from the absorbed O_2_ or dissolved O atoms. This interfacial layer tightly binds the graphene to the underlying SiC and enhances structural integrity. Such strong interfacial bonding provides efficient electronic pathways and accelerates Li^+^ transport across the heterointerface, which is critical for achieving high charge-transfer kinetics and stable electrochemical behavior [33]. Overall, the Si 2p and C 1s spectral features correspond to the silicon core, SiC interlayer, and epitaxial graphene shell, unambiguously confirming the successful construction of the Si@SiC@EG heterostructure.

**Figure 3:**
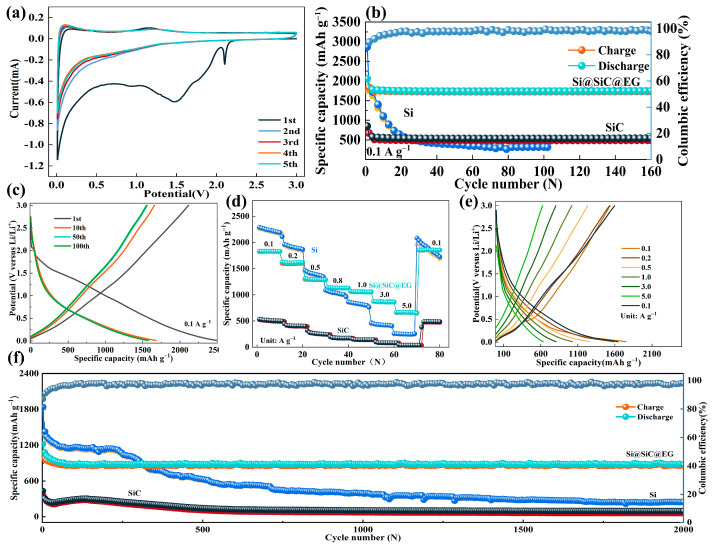
(**a**) CV curves at 0.1 mV s^−1^. (**b**) Compared cycling performances at 0.1 A g^−1^. (**c**) Charge/discharge profiles of Si@SiC@EG at 0.1 A g^−1^. (**d**) Comparison of the specific capacities at 0.1, 0.2, 0.5, 0.8, 1.0, 3.0, 5.0 and 0.1 A g^−1^. (**e**) Charge/discharge profiles at 0.1, 0.2,0.5, 1.0, 3.0, 5.0 and 0.1 A g^−1^. (**f**) Compared long-cycling performance at 1.0 A g^−1^.

All electrochemical data presented in Figure 3 are obtained using CR2032-type coin half-cells, with Si@SiC@EG as the working electrode and lithium metal as the counter and reference electrode. Cyclic voltammetry (CV) tests are employed to understand the lithiation/delithiation process of Si@SiC@EG anode. At 0.1 mV s^−1^, the CV curves are similar to those reported for SiC anodes (Figure 3a), indicating that the lithium-ion storage mechanism of Si@SiC@EG is dominated by the process of lithium-ion insertion/desertion in SiC, and the encapsulation of EG has no substantial interference with the lithium-ion storage mechanism. As shown in Figure 3a, within the voltage range of 0–3 V, the Si@SiC@EG anode exhibits irreversible reduction peaks at approximately 2.1, 1.5, and 1.0 V in the first cycle, corresponding to electrolyte decomposition, SEI formation, and side reactions between EG, SiC, and Si with the electrolyte [34,35]. In subsequent cycles, the nearly overlapping CV curves indicate a highly reversible lithiation/delithiation process, confirming the excellent electrochemical reversibility and structural stability of the Si@SiC@EG anode [36]. The cycling behavior of the Si, SiC, and Si@SiC@EG anodes at a current density of 0.1 A g^−1^ is illustrated in Figure 3b. Among the three, the Si@SiC@EG anode delivers the high initial discharge capacity and maintains excellent structural stability throughout prolonged cycling, retaining a reversible capacity of 1747 mAh g^−1^ after 160 cycles. A modest capacity decline occurs during the early cycles, which is primarily associated with the irreversible consumption of lithium during solid electrolyte interphase (SEI) formation and gradual anode activation [37]. After this initial stabilization, the capacity remains highly stable, indicating the effectiveness of the core–shell architecture in mitigating volume fluctuations and sustaining long-term electrochemical performance.

After the initial activation stage, the specific capacity gradually stabilizes, accompanied by a steady increase in coulombic efficiency (CE). Within 20 cycles, the CE rises to 99.3% and remains at this level throughout subsequent cycling, reflecting excellent interfacial stability and highly reversible electrochemical behavior. In comparison, the pristine Si anode undergoes rapid degradation, with a pronounced drop occurring within the first 20 cycles. This rapid fading originates from the drastic volume expansion of Si during repeated lithiation/delithiation, which causes particle pulverization, the loss of electrical contact, and unstable SEI layer growth [38,39,40]. Consequently, its capacity falls to approximately 320 mAh g^−1^ after 100 cycles. The SiC anode, in contrast, shows the lowest initial capacity but remarkable long-time cycling stability, delivering 543 mAh g^−1^ after 160 cycles. This behavior reflects the inherent structural rigidity of SiC and its limited lithiation capacity. Notably, the Si@SiC@EG composite inherits the high theoretical capacity of Si while leveraging the mechanical resilience of SiC and the excellent electronic conductivity of epitaxial graphene. This synergistic configuration effectively mitigates stress arising from Si expansion and preserves stable electron/ion transport pathways, resulting in both high capacity and extended cycling life. The cycling test at 0.1 A g^−1^ is intentionally terminated after 160 cycles as part of our predefined experimental protocol, since the long-term cycling stability is evaluated separately at 1 A g^−1^ over 2000 cycles (Figure 3f). The charge/discharge profiles in Figure 3c is recorded during the initial cycles of the same sample shown in Figure 3b. The higher capacity observed in the early cycles is due to SEI formation and initial activation, which typically results in higher first-cycle capacities compared to the stabilized values shown in Figure 3b. The first discharge and charge capacities reach 2489 mAh g^−1^ and 2115 mAh g^−1^, respectively, corresponding to an initial coulombic efficiency (ICE) of 84.97%. This high ICE is closely related to the double-shell structure, which promotes electrochemical stability. Moreover, the improvement compared to Si and SiC nanoparticles can be linked to enhanced interfacial electron migration and charge-transfer kinetics driven by the built-in electric field [41]. The nearly overlapping GCD curves during repeated cycling further confirm the Si@SiC@EG anode’s outstanding cycling stability and stable architecture, consistent with the CV observations. The rate capability of the three anodes is evaluated from 0.1 to 5.0 A g^−1^ current densities (Figure 3d). Si@SiC@EG clearly outperforms both pristine Si and SiC. It delivers a reversible capacity of 1856 mAh g^−1^ at 0.1 A g^−1^ and maintains a large fraction of its capacity with the current increases. Even at 5.0 A g^−1^, the anode retains considerable capacity, highlighting its excellent rate performance under fast charge/discharge conditions. When the current is switched back to 0.1 A g^−1^, the capacity almost completely recovers, indicating strong structural reversibility during high-rate conditions. In contrast, the Si anode exhibits a steep capacity decline at elevated rates, mainly due to sluggish Li^+^ diffusion and structural damage during charge/discharge progress [42]. Although the SiC anode remains mechanically stable, its intrinsic capacity is limited by the low lithiation ability of SiC [43]. The outstanding rate performance of Si@SiC@EG stems from its hierarchical structure: the in situ-grown SiC layer accommodates mechanical strain and preserves particle integrity, while the epitaxial graphene coating establishes a continuous conductive network, accelerates charge transfer, and minimizes polarization under demanding conditions. The corresponding GCD curves at different current densities (Figure 3e) further underscore the excellent kinetic behavior of this composite anode. These results of rate collectively suggest that the double encapsulation may enhance the electrolyte wettability and provides a multitude of optimal sites for lithium-ion insertion, thereby improving both energy density and electrochemical kinetics. The long-term stability of the Si@SiC@EG anode is further evaluated at a current density of 1.0 A g^−1^. As shown in Figure 3f, the reversible capacity remains essentially unchanged during the first 200 cycles, approaching nearly 100% retention and demonstrating exceptional cycling durability. After completion of 2000 charge/discharge cycles, the reversible capability of 872 mAh g^−1^ is achieved, representing approximately 73.56% of the retained second discharge capacity (estimated at 1186 mAh g^−1^). The average capacity fading is about 1.32% per cycle. For the SiC NPs and Si NPs, the reversible capacities after 2000 cycles are 97 mAh g^−1^ and 248 mAh g^−1^ at 1.0 A g^−1^, respectively. The capacity retention can reach 25.52% and 13.50% of the 2nd discharge capacity (~380 mAh g^−1^ for SiC NPs, ~1837 mAh g^−1^ for Si NPs), respectively. The average capacity fading is about 3.73% and 4.32%, respectively. The outstanding lithium-ion storage performance of the Si@SiC@EG anode stems from the cooperative contributions of its well-designed architecture. (i) The heterostructure facilitates the formation of a uniform and stable SEI layer, which reduces continuous electrolyte decomposition and limits the generation of inactive lithium [44,45]. (ii) The in situ-grown SiC layer functions as a mechanical buffer that relieves stress from repeated volume fluctuations of the silicon core, thereby maintaining the structural integrity of the anode. (iii) The epitaxial graphene coating provides a continuous conductive network while acting as a flexible and chemically stable barrier, ensuring close interfacial contact and enabling rapid charge transfer. (iv) In addition, the strong Si-C interfacial bonding strengthens the overall framework of the composite, helping to prevent active material fracture and preserve anode integrity during prolonged cycling [46]. This integrated structural design enables Si@SiC@EG to combine the high theoretical capacity of Si with the mechanical stability of SiC and the superior electrical conductivity of graphene, thereby achieving both high capacity and excellent cycling durability. A broader comparison of our Si@SiC@EG anode with previously reported Si-based anodes is summarized in Table S1 (Supporting Information). The table highlights that the present material offers a favorable balance of high capacity, long-cycle durability, and practical synthesis, demonstrating competitive advantages relative to other core–shell or Si@C-based architectures.

**Figure 4:**
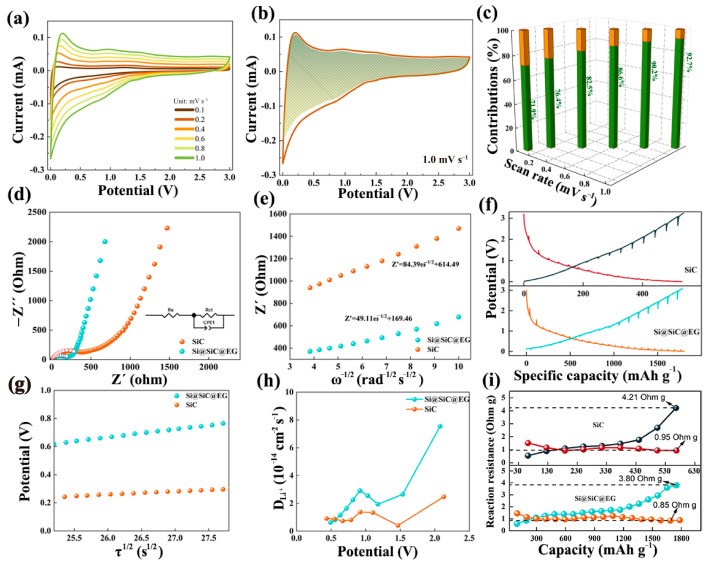
(**a**) CV curves at different scan rates. (**b**) The calculated surface and diffusion capacitance at 1.0 mV s^−1^. (**c**) The relative proportions of capacitance and diffusion-controlled capacity at different scan rates. (**d**) EIS of Si@SiC@EG and SiC NPs, the illustration is electrical circuit model. (**e**) The relationships between *Z*′ and *ω*^1/2^. (**f**) GITT curves of Si@SiC@EG and SiC NPs. (**g**) Linear relation between *V* and *τ*^1/2^. (**h**) Lithium-ion diffusion coefficient and (**i**) reaction internal resistance during the lithiation/delithiation.

The electrochemical kinetics of the Si@SiC@EG anode are examined through CV measurements at different scan rates (Figure 4a). The CV profiles exhibit distinct redox peaks with only slight shifts, reflecting a highly reversible lithiation/delithiation process and rapid charge-transfer dynamics. To gain further insight into the storage mechanism, the relationship between peak current (*i*) and scan rate (*v*) is analyzed using the power-law equation:

$$i=a\;v^{b}$$

where *i* and *v* are the current density and the potential scan rate, respectively. *a* and *b* are both adjustable parameters. The *b*-value approaches 0.5 is characteristic of diffusion processes, while *b*-value approaches 1.0 indicate surface-capacitive processes. The linear fit of log(*i*) versus log(*v*) gives b values consistently above 0.5 for all characteristic peaks (Figure S3), revealing a mixed contribution of both diffusion and surface-capacitive processes, with the latter making a substantial contribution to charge storage [47]. The capacitive component is quantified by separating the current response according to *i* = *k*_1_*v* + *k*_2_*v*^1/2^, where the first term corresponds to the capacitive effect and the second to diffusion-controlled insertion. As shown in Figure 4b, the pseudocapacitive fraction reaches 92.7% at 1.0 mV s^−1^, underscoring the strong capacitive contribution to the overall charge-storage behavior [48]. This pronounced capacitive response can be linked to the distinctive double-shell design: the epitaxial graphene shell offers a highly conductive matrix, while the SiC layer improves interfacial stability and maintains efficient ion transport pathways. The evolution of pseudocapacitance with increasing scan rates is presented in Figure 4c. Even under high-rate conditions, the capacitive fraction remains dominant, reflecting the rapid and reversible interfacial reaction characteristics of the composite anode. This behavior arises from the abundance of heterogeneous interfaces within the structure, which provide numerous active sites, may enhance electrolyte wettability, and accelerate interfacial charge transfer [49]. Furthermore, the mechanically resilient double-shell framework accommodates repeated volume fluctuations, preserving anode integrity during extended cycling [50]. The pseudocapacitive-dominated mechanism thus enables ultrafast ion and electron transport, laying the foundation for the excellent rate performance and cycling stability of the Si@SiC@EG anode.

As shown in Figure 4d, the Si@SiC@EG and SiC anodes exhibit typical Nyquist profiles characterized by a depressed semicircle in the high-to-medium frequency region followed by an inclined line at low frequencies. The former corresponds to the charge transfer resistance (*R_ct_*), while the latter reflects the diffusion process of lithium-ion at the interface. A clear reduction in *R_ct_* is observed for Si@SiC@EG (204.8 Ω) compared to the SiC anode (287.5 Ω), demonstrating that the graphene shell significantly facilitates interfacial electron migration and accelerates charge-transfer kinetics. To further quantify the diffusion behavior, the real part of the impedance is analyzed using the equation:

$$Z_{real}\;=R_{e}+R_{ct}+\sigma \omega^{-1/2}$$

where σ is the Warburg factor and ω is the angular frequency. As shown in Figure 4e, the calculated σ value for the Si@SiC@EG anode is 49.11, lower than that of the SiC anode (84.39) [51]. A smaller σ reflects faster Li^+^ diffusion within the anode, consistent with the reduced interfacial resistance [52]. This improvement arises from the intimate contact between the EG shell and the SiC layer, which shortens the electron pathways and enables more efficient ion migration. The combination of fast charge transfer and improved diffusion kinetics provides a strong foundation for the excellent rate capability of the Si@SiC@EG anode.

The electrochemical kinetics of the Si@SiC@EG composite are investigated by the galvanostatic intermittent titration technique (GITT). A current pulse of 0.1 A g^−1^ for 20 min is applied to measure the closed-circuit voltage (CCV), followed by a 40 min relaxation period to record the quasi-open-circuit voltage (QOCV). To minimize the influence of SEI formation, the GITT measurement is conducted after 10 activation cycles. In the comparison of Figure 4f, the lower overpotential shows the fast electrochemical kinetics of the Si@SiC@EG anode. The lithium-ion diffusion coefficient (*D_Li_*^+^) can be calculated according to Fick’s second law:

(1) $$D_{{\mathrm{Li}}^{+}}=\frac{4}{\pi }{\left(\frac{m_{B}V_{B}}{M_{B}S}\right)}^{2}{\left(\frac{∆E_{s}}{\tau \left(\frac{dE_{\tau }}{d\sqrt{\tau }}\right)}\right)}^{2}\left(\tau \ll \frac{L^{2}}{D_{{\mathrm{Li}}^{+}}}\right)$$

where *m_B_* (g) and *M_B_* (g mol^−1^) are the active mass and molecular weight of SiC NPs or Si@SiC@EG; A (cm^2^) is the anode/electrolyte contact area; *τ* (s) is the pulse time; Δ*E_τ_* (V) is the voltage change of single step GITT; and Δ*E_s_* (V) is the steady-state voltage change between steps [53]. The conclusion that the square root of the pulse duration (*τ*^1/2^) is linearly related to the potential can be obtained in Figure 4g, thus allowing *D_Li_*^+^ to be further calculated. In Figure 4h, the trends of *D_Li_^+^* are similar in SiC NPs and Si@SiC@EG, which indicates that the diffusion behavior of lithium-ion is consistent. The Si@SiC@EG have an overall superior *D_Li_*^+^, showing alleviation of polarization effects and improvement of lithium-ion reaction kinetics. Furthermore, the reaction resistance can be acquired according to CCV and QOCV.

As shown in Figure 4i, the Si@SiC@EG anode exhibits a markedly lower reaction resistance, reflecting faster lithium-ion transport and more accessible active sites provided by the graphene encapsulation. Compared with the pristine SiC nanoparticle anode, it delivers not only a higher specific capacity but also more efficient charge-transfer behavior and a larger D_Li_^+^ value, pointing to significantly improved reaction kinetics.

**Figure 5:**
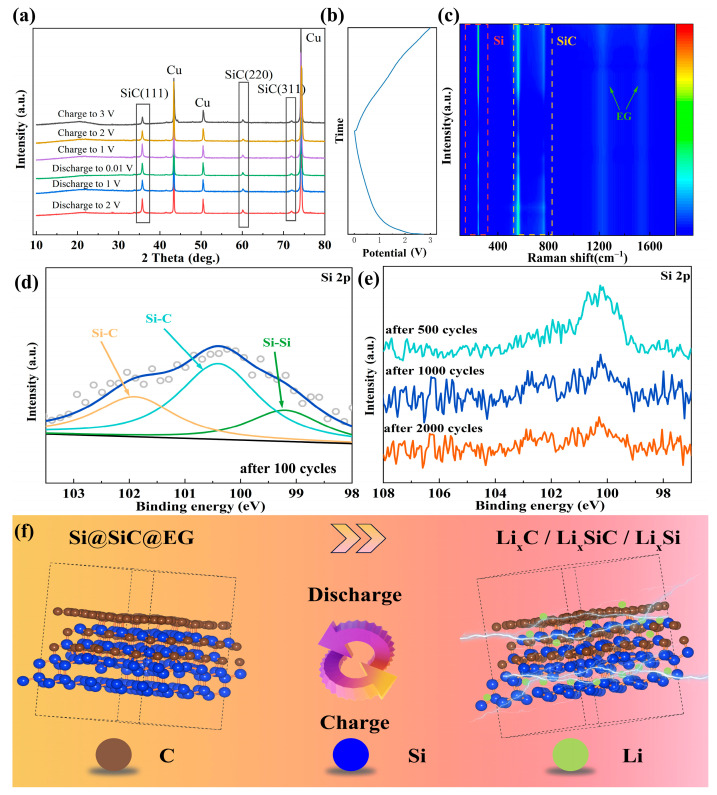
(**a**) Ex situ XRD plot of Si@SiC@EG. (**b**) The GCD curve during the in situ Raman testing. (**c**) In situ Raman plot and (**d**,**e**) Ex situ XPS analysis of the Si@SiC@EG anode. (**f**) Schematic diagram of lithium-ion storage process in the Si@SiC@EG anode.

To elucidate the structural evolution of the Si@SiC@EG anode during cycling, ex situ XRD, XPS, and in situ Raman analyses are carried out. As shown in Figure 5a, the XRD patterns collected at different charge and discharge states remain essentially unchanged, with all characteristic peaks of SiC clearly retained throughout the process. No additional phases appear, only changes in intensity. This behavior points to a highly reversible lithiation/delithiation process dominated by Li^+^ insertion and extraction, rather than alloying-induced phase transformation. The corresponding charge/discharge curve used during the in situ Raman measurements is presented in Figure 5b, which correlates the potential states with the Raman spectral evolution shown in Figure 5c. This provides a direct linkage between the structural transitions and the electrochemical states during lithiation and delithiation. The evolution of the Raman signal in the range of 100–1800 cm^−1^ is shown as a contour plot in Figure 5c. The characteristic silicon peak at approximately 202 cm^−1^, together with the SiC-related features near 462 and 627 cm^−1^, gradually weakens upon discharge [54,55]. This change reflects lithium insertion and the formation of Li_x_Si and Li_x_SiC intermediates. When the cell is recharged, these peaks largely reappear, highlighting the reversibility of the lithiation/delithiation process. The persistence of Si and SiC signatures throughout the entire cycle implies that the double-shell architecture effectively relieves internal stress, maintains a continuous conductive framework, and keeps ion transport pathways open. Combined ex situ XRD and in situ Raman analyses thus provide direct structural support for a fully reversible lithium insertion/extraction mechanism in Si@SiC@EG. In contrast to conventional Si anodes that often amorphize and fracture, this engineered heterostructure preserves both crystallinity and interfacial stability, which is consistent with its superior electrochemical durability.

Ex situ XPS is further employed to examine the chemical environment of Si after 100 charge/discharge cycles (Figure 5d). The Si 2p spectrum shows three components associated with Si-Si and Si-C bonds. While the overall intensity slightly decreases compared to the original anode (Figure 2c), the binding energies of the Si-C (~100.3 and ~101.5 eV) and Si-Si (~99.3 eV) components remain unchanged, indicating a stable chemical environment during long-term cycling. Extended cycling tests (500, 1000, and 2000 cycles) yield similar results (Figure 5e). This stability suggests that the Si core is effectively shielded from oxidation and irreversible phase transitions, even under prolonged operation. Such preservation of the Si 2p signal confirms that the composite structure suppresses interfacial degradation and side reactions, thereby sustaining a stable electronic environment and ensuring long-term reversibility.

The lithium-storage mechanism is illustrated schematically in Figure 5f. Initially, the anode consists of a crystalline Si core, an in situ-formed SiC interlayer, and an outer graphene shell. During lithiation, Li^+^ ions first intercalate into the graphene layer (forming Li_x_C), diffuse through the SiC layer (forming metastable Li_x_SiC), and finally alloy with the Si core to generate Li_x_Si, which provides the primary capacity. The SiC layer serves both as a mechanical buffer and a chemical barrier, accommodating the volume expansion of Si while limiting side reactions at the anode-electrolyte interface [56]. Meanwhile, the graphene framework delivers efficient electron conduction and mechanical flexibility, further enhancing interfacial kinetics. Upon delithiation, Li^+^ ions are extracted from all phases in a highly reversible manner, and the structural framework is restored without collapse. This mechanism ensures efficient charge transport, minimal interface degradation, and excellent capacity retention during long-term cycling.

**Figure 6:**
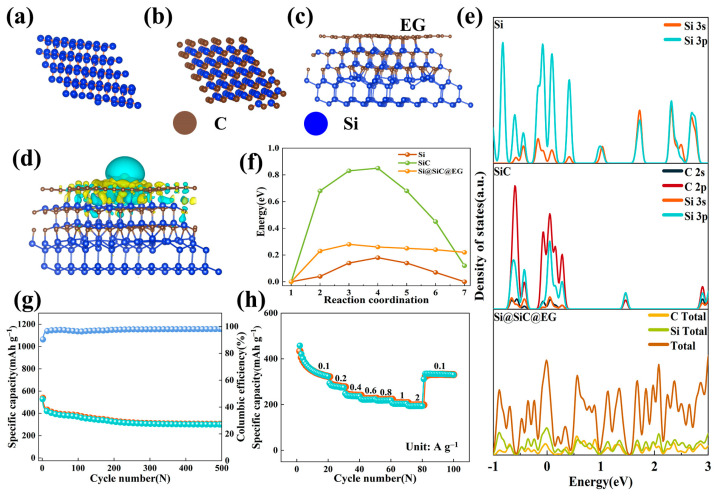
(**a**–**c**) The atomic structure models of the Si, SiC, and Si@SiC@EG. (**d**) Charge density plot of the Si@SiC@EG heterostructure, light blue and yellow regions represent the isosurfaces of electron depletion and accumulation. (**e**) Calculated total density of states (DOS) of the Si, SiC, and Si@SiC@EG. (**f**) Plot of Li^+^ migration energy barrier in Si@SiC@EG heterostructure. (**g**) Electrochemical performance test plots of full cells at 0.1 A g^−1^. (**h**) Rate performance of full cells from 0.1 A g^−1^ to 2.0 A g^−1^.

To gain atomic-level insight into the interfacial electronic behavior, density functional theory (DFT) calculations are performed based on the structure-optimized models of Si, SiC, and the Si@SiC@EG heterostructure. As shown in Figure 6c, the Si-C bonding at the interfaces between Si and SiC, as well as between SiC and epitaxial graphene, is thermodynamically robust, consistent with the interfacial bonding features observed experimentally by XPS. Such strong interfacial coupling is expected to provide a stable framework for efficient electron transport during cycling. The charge density difference calculations reveal a distinct redistribution of electrons at the heterointerface (Figure 6d). Pronounced charge accumulation around the Si-C bonding regions indicates stronger orbital interactions between Si and C, which promote electron delocalization and reduce interfacial resistance. This enhanced electronic coupling plays a central role in accelerating charge-transfer processes within the composite. The influence of this interface on the electronic structure is further captured in the calculated density of states (DOS) profiles (Figure 6e). Whereas pristine Si and SiC exhibit semiconducting features with clear band gaps, the DOS of Si@SiC@EG displays a downward shift of the conduction band across the Fermi level, characteristic of a metallic-like transition [57]. As shown in Figure 6f, the Li^+^ migration barrier within the Si@SiC@EG heterostructure is significantly lower than that of pristine Si and SiC. This reduction arises from the strong interfacial Si-C coupling and the presence of the conductive epitaxial graphene pathway, which together form energetically favorable Li^+^ diffusion channels across the interface [58]. The calculated barrier is consistent with the experimentally observed enhancement in charge-transfer kinetics and diffusion behavior (Figure 4e–i). This improvement originates from the combined effect of the conductive graphene shell and the chemically bonded SiC interface, which together create interconnected pathways for both electron and ion movement. These theoretical insights are consistent with the electrochemical results, underscoring the crucial role of interfacial engineering in enabling fast, efficient charge and ion transport in the Si@SiC@EG anode.

The electrochemical performance test results of the Si@SiC@EG anode show that its electrochemical performance in the half-cell is excellent (Figure 3). To further evaluate its practical application feasibility, it is assembled into a full cell for evaluation. Si@SiC@EG is used as the anode of the assembled full cell, LiFePO_4_ as the cathode, and the capacity ratio of the anode to the cathode is 1.1:1. As shown in Figure 6g, at a current density of 0.1 A g^−1^, after 500 cycles, its CE remained stable at 98.13%, and after 300 cycles at this current density, the discharge capacity is 306 mAh g^−1^. Moreover, the GCD curve fitting is good after 300 cycles (Figure S4), indicating that the full cell had extremely high cycle reversibility at a current density of 0.1 A g^−1^ during the test. The full-cell capacity is significantly lower than the theoretical value of the Si@SiC@EG anode due to several practical constraints. The lithiation depth of the anode is limited by the LiFePO_4_ cathode, which provides a restricted Li^+^ reservoir compared with the unlimited lithium supply in half-cells. The N/P ratio of 1.1:1 further constrains the accessible capacity and prevents deep lithiation of silicon to avoid instability. Additionally, electrode-level factors such as mass loading and kinetic polarization contribute to the reduced practical capacity in the assembled full cell. As shown in Figure 6h, the cycle performance of the full cell is also excellent under different rates. At current densities of 0.1, 0.2, 0.4, 0.6, 0.8, 1, 2, and 0.1 A g^−1^, the discharge capacities are 323.4, 274.4, 236.3, 222.5, 218.2, 193.78, and 330.18 mAh g^−1^, respectively. Its outstanding lithium-ion reaction kinetics are the main reason for the excellent rate performance of the full cell. The test results of Si@SiC@EG in the full cell indicate that it performs well in terms of cycle stability and rate performance. Therefore, Si@SiC@EG as the anode of the full cell has sufficient application potential.

## 3. Conclusions

In conclusion, a Si@SiC@EG double-shell heterostructure has been successfully developed through interfacial engineering. The in situ-grown SiC layer, together with the epitaxial graphene coating, creates strong Si-C bonds and continuous pathways for both electron and ion transport. This hierarchical design effectively accommodates the volume fluctuations of silicon and stabilizes the SEI layer, which can improve the performance of conventional Si anodes. Benefiting from this architecture, the Si@SiC@EG anode delivers a reversible capacity of 1747 mAh g^−1^ after 160 cycles at 0.1 A g^−1^ and maintains the reversible capacity of 872 mAh g^−1^ after 2000 cycles at 1 A g^−1^. In situ and ex situ spectroscopic analyses reveal a highly reversible Li^+^ insertion/extraction process, while theoretical calculations point to lower Li^+^ migration barriers at the engineered interface. When assembled in a full cell with a LiFePO_4_ cathode, the system sustains a discharge capacity of about 300 mAh g^−1^ after 500 cycles, highlighting its practical applicability. This design concept provides a promising direction for advancing silicon-based anodes for high-energy, long-life lithium-ion batteries.

## Data Availability

The original contributions presented in this study are included in the article/Supplementary Material. Further inquiries can be directed to the corresponding authors.

## Supplementary Materials

The following supporting information can be downloaded at: https://www.mdpi.com/article/10.3390/molecules30234517/s1, Supporting Information S1: Experimental details; Supporting Information S2: Characterization methods; Supporting Information S3: Electrochemical measurements; Supporting Information S4: Density functional measurements [59,60,61,62,63]; Supporting Information S5: Results and discussion; Figure S1: (a) FFT of Si(111). (b) FFT of SiC(111); Figure S2: XRD spectrum in 20–25°; Figure S3: b value derived from the CV curves; Figure S4: Charge/discharge profiles of full cells at 0.1 A g^−1^; Table S1: Comparisons of the synthetic method, morphology, cycle number, current density, and capacity between the Si@SiC@EG anode and other previously reported Si-based LIBs anodes [64,65,66,67,68,69,70,71,72,73].
