# Supplementary material for: Interfacial Electronic Coupling in Si@SiC@EG Core–Shell Architectures Enables High-Capacity and Long-Life Lithium-Ion Batteries
Source: Molecules. 2025 Nov 22;30(23):4517. doi: 10.3390/molecules30234517 (PMC12693415; doi:10.3390/molecules30234517)
Supplement: Supplementary file 1 [file molecules-30-04517-s001.zip › molecules-3982901-supplementary.pdf]

Interfacial Electronic Coupling in Si@SiC@EG Core-Shell Architectures Enables High-Capacity and Long-Life Lithium-Ion Batteries

Huangyu Zhao[1,2,5], Sihao He[1,5], Changlong Sun[5], Kesheng Gao[2], Honglin Li[3], Qiuju Zheng[4], Lingshan Geng[5], Yan-Jie Wang[1]*, Enyue Zhao[2]*, Yuanyuan Zhu[5]*

[1] New Energy and Advanced Functional Materials Group, School of Materials Science and Engineering, Dongguan University of Technology, Dongguan 523808, Guangdong, P. R. China

[2] Songshan Lake Materials Laboratory, Dongguan 523808, Guangdong, P. R. China

[3] College of Materials Science and Engineering, Qingdao University of Science and Technology, Qingdao 266042, Shandong, P. R. China

[4] School of Materials Science and Engineering, Qilu University of Technology, Jinan, 250353, Shandong, P. R. China

[5] Key Laboratory of Spin Electron and Nanomaterials of Anhui Higher Education Institutes, Suzhou University, Suzhou 234000, P. R. China.

* Corresponding authors E-mail:

wyj@dgut.edu.cn (Yan-Jie Wang)

eyzhao@sslab.org.cn (Enyue Zhao)

zhuyy@ahszu.edu.cn (Yuanyuan Zhu)

Contents:

S1. Experimental details

S2. Characterization methods

S3. Electrochemical measurements

S4. Density functional theory calculation

S5. Results and discussion

Figure S1. (a)FFT of Si(111) (b)FFT of SiC(111).

Figure S2. XRD spectrum in 20°-25°.

Figure S3. b value derived from the CV curves.

Figure S4. Charge/discharge profiles of full cells at 0.1 A g^{-1}.

Table S1. Comparisons of the synthetic method and electrochemical performance between the Si@SiC@EG electrode and other previously reported Si-based LIBs electrodes

S6. References

S1. Experimental details

Synthesis of Si@SiC@EG Composite

The raw Si particles (Macklin, Inc., D50 ≈ 30 nm) were dispersed in acetone via ultrasonic treatment for 15 min to obtain a homogeneous suspension. Poly (ethylene glycol) diacrylate (PEGDA, Macklin, Inc.) was similarly dissolved in acetone and ultrasonicated for 15 min to form a uniform solution. The two solutions were then mixed, followed by the addition of azobisisobutyronitrile (AIBN, Macklin, Inc.). The precursor mixture consisted of carbon precursor : Si : additive in a mass ratio of 8 : 1.8 : 0.2. The mixture was ultrasonicated for an additional 30 min and subsequently stirred until complete evaporation of the acetone. The resulting oligomer was thermally cured at 70 °C to ensure full polymerization, yielding the Si@PEO composite.

The dried Si@PEO was transferred to an alumina crucible and placed in a tube furnace. The chamber was evacuated to ≤ 1 mTorr using a vacuum pump and purged with high-purity argon three times to remove residual air. The dried Si@PEO was transferred to an alumina crucible and placed in a tube furnace. After evacuation to ≤ 1 mTorr and three argon purge cycles, the sample underwent the first thermal treatment to form Si@SiC. The temperature was increased to 1000 °C at a heating rate of 5 °C min^{-1}, followed by a slower ramp from 1000 °C to 1450 °C at 2 °C min^{-1}. The sample was maintained at 1450 °C for 120 min under an argon atmosphere (100 kPa). Afterward, the temperature was decreased to 1000 °C at 5 °C min^{-1}, and the system was then allowed to cool naturally to room temperature.

After natural cooling to room temperature, the Si@SiC particles were collected,

ground for 15 min, and cleaned via ultrasonic washing with deionized water and absolute ethanol for 30 min. The washed particles were dried at 80 °C in an oven. Subsequently, the Si@SiC powder was loaded into a graphite crucible and placed back into the tube furnace. After evacuation to ≤ 1 mTorr and three argon purge cycles, the sample was subjected to a second thermal treatment to obtain Si@SiC@EG particles. The furnace temperature was increased to 1000 °C at a heating rate of 5 °C min^{-1}, followed by a slower ramp from 1000 °C to 1500 °C at 2 °C min^{-1}. After maintaining 1500 °C for 15 min under an argon atmosphere (100 kPa), the system was cooled to 1000 °C at 5 °C min^{-1}, and then allowed to cool naturally to room temperature. The final Si@SiC@EG product was collected after cooling.

Electrode Fabrication

The slurry is prepared by mixing Si@SiC@EG : Super P : PVDF = 8 : 1 : 1 (wt%) in N-methyl-2-pyrrolidone (NMP) to form a uniform dispersion. The slurry was coated onto copper foil using a doctor blade with a gap of 100 μm at a coating speed of 5 mm s^{-1}. The coated electrodes were dried at 80 °C for 12 h under vacuum. After drying, the electrodes were calendared under a pressure of 20 MPa to achieve uniform density. The average electrode thickness (~50 μm) was measured using a digital micrometer, and the active material loading (~1.2 mg cm^{-2}) was determined by weighing the electrodes before and after coating. Circular electrodes were punched and used for subsequent electrochemical measurements.

S2. Characterization methods

The structure and morphology of the pristine Si@SiC@EG nanoparticles were

characterized by scanning electron microscopy (SEM, Hitachi S-4800). Energy-dispersive spectroscopy (EDS) elemental scans were performed on the same instrument equipped with a detector (7593-H, Horiba). The powder X-ray diffraction (XRD) measurements were performed on a Rigaku D/MAXRB diffractometer (Philips, X'pert Pro MPD, Netherlands), and the excitation wavelength is Cu Kα radiation (λ =0.15443 nm). Transmission electron microscopy (TEM) and corresponding high-resolution images were obtained using a Philips Tecnai 20U-Twin microscope at an acceleration voltage of 200 kV. As for TEM sample preparation, the powder was dispersed in ethanol, and then dropped on a copper micro-grid covered by a carbon film. The specimen for TEM was set statically and dried naturally in air before its characterization. The room-temperature Raman spectra were measured with 532 nm photons from an Ar^+ laser. The characteristics of the graphene formed on the surface of the Si@SiC powder were analyzed using the linearly-polarized light, which was focused onto the sample through a 100× optical microscope objective with a spatial resolution of < 2 μm (in diameter) in a backscattering geometry. The laser power used was < 1.5 mW. The binding energies of the pristine Si@SiC@EG nanoparticles were acquired by X-ray photoelectron spectroscopy (XPS) on the Thermo ESCALAB 250 with Al Kα radiation (1486.8 eV) as the excitation source.

S3. Electrochemical measurements

The electrochemical behaviors of the pristine Si@SiC@EG nanoparticles were investigated by assembling CR2032-type coin cells, which were assembled in an argon-

filled glove box with a concentration of moisture and oxygen below 1 ppm. During the preparation of the pristine Si@SiC@EG working electrodes of the tested cells, 80 wt% active materials, 10 wt% conductive carbon (ketjen black), and 10 wt% polyvinylidene fluoride (PVDF) as the binder were mixed in N-methyl-2-pyrrolidone, then the well-mixed active materials were pasted on copper foil, and then dried at 80 °C overnight under vacuum before use. The average loading density of these active materials was about 1.9 mg with a diameter of 12 mm. Li metal as both the counter and reference electrodes. The positive and negative electrodes were electronically separated by a polypropylene film (Celgard 2500) saturated with electrolyte. The electrolyte solution was $LiPF_6$ (1 M) in ethylene carbonate/dimethyl carbonate/diethyl carbonate (1:1:1 vol%). A Neware CT-3008W battery measurement system (Neware Technology Ltd., P. R. China) was used to carry out galvanostatic charge and discharge tests at various current densities with a cut-off voltage of 0.01~3.0 V vs. Li^+/Li, at room temperature. The galvanostatic intermittent titration technique (GITT) was tested on a Neware battery tester as well. The cell was discharged at 0.2 A g^{-1} for 5 min, followed by a 20 min relaxation, within the voltage window of 0.01 ~ 2.8 V. The cyclic voltammetry (CV) was performed at different scan rates within the range of 0.01-3.0 V using a CHI660D electrochemical workstation (Shanghai CH Instruments Co., China). Electrochemical impedance spectroscopy (EIS) measurements were also carried out on the CHI660D electrochemical workstation over frequencies ranging from 0.01 Hz to 1 MHz at a 5 mV amplitude signal and no applied voltage bias. After cycling, the Si@SiC@EG electrodes were taken from the sealed cells for *ex-situ* XRD and XPS measurement, and

then the Si@SiC@EG electrodes were soaked in DEC for 1 h, then thoroughly washed with diethyl carbonate, vacuum-dried in the argon-filled glove box, and transferred to the spectrometer using a sealed transport bag to avoid oxidation. The cycled Si@SiC@EG powders were transferred to the TEM holder with minimal ambient exposure (<5 min).

The Li-ion full cell was fabricated by using a commercial $LiFePO_4$ cathode (70 wt% active material, 20 wt% carbon black, and 10 wt% PVDF) and the Si@SiC@EG anode. In order to alleviate the formation of lithium dendrites during cycling, the capacity ratios of anode and cathode were controlled around 1.1 : 1. The calculation process is as follows:

$$\frac{C_{\text{anode}} \times m_{\text{anode}}}{C_{\text{cathode}} \times m_{\text{cathode}}} = 1.1: 1$$

where C is the specific capacity, m is the loading mass.

The mass loading of the Si@SiC@EG anode is 1.9 mg. At 0.1 A g^{-1}, C_{anode} is 1740 mA h g^{-1}, C_{cathode} is 155 mA h g^{-1}. Therefore, the mass loading of the $LiFePO_4$ cathode is 19.39 mg, and the calculated mass ratio of the anode and cathode was set at 1 : 10.2.

S4. Density functional theory calculation

First-principal calculations were performed using the Vienna Ab Initio Simulation Package [1] within the projector augmented-wave approach. The generalized gradient approximation (GGA) exchange-correlation function developed by Perdew, Burke, and Ernzerhof [2] was used for the exchange-correlation potential. The energy cutoff for plane-wave expansion was set to 440 eV. All the structures were relaxed until the forces

became less than 0.01 eV/Å, and the energy tolerances were less than 10^{-5} eV/atom. The DFT + U method was used with the Dudarev approach [3] implemented in the Vienna Ab Initio Simulation Package, where U is the on-site Coulomb parameter to calculate the average voltages of Li intercalation. The K-point of the Brillouin zone was sampled using a 3 × 3× 1 gamma-centered Monkhorst–Pack grid for the unit cell. A vacuum of 20 Å between the layers was considered to safely avoid the interaction between the periodically repeated structures. We used the climbing image nudged elastic band method (CI-NEB) [4] to determine the energy barriers and minimum energy paths of Li diffusion [5]. Each transition state showed only one imaginary frequency, and the vibration mode displayed the right path connecting the reactant and product.

S5. Results and discussion

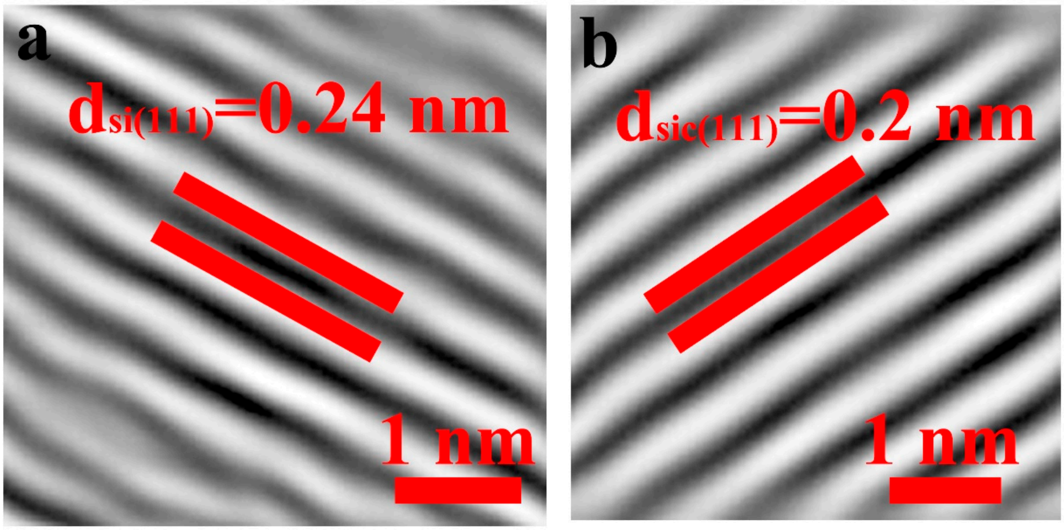

Figure S1. (a)FFT of Si(111) (b)FFT of SiC(111).

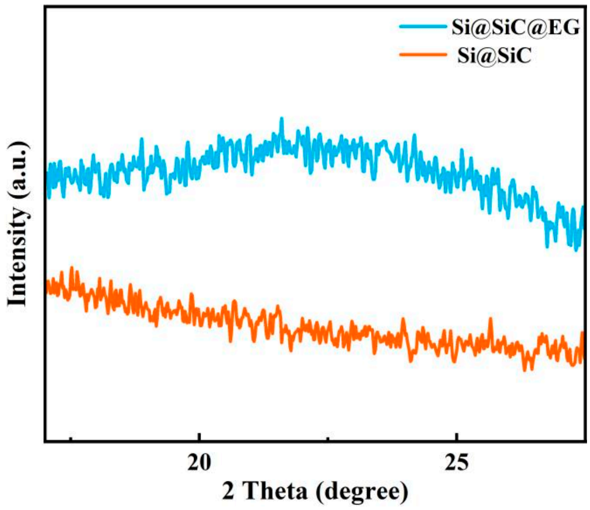

Figure S2. XRD spectrum in 20°-25°.

The enlarged spectrogram of Si@SiC@EG within the range of 20°-25°.

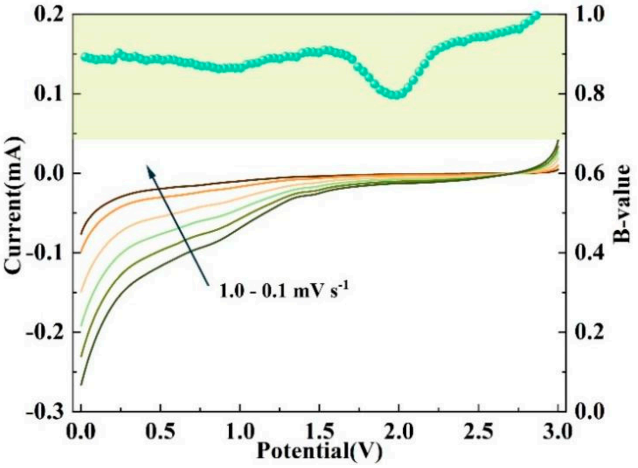

Figure S3. b value derived from the CV curves.

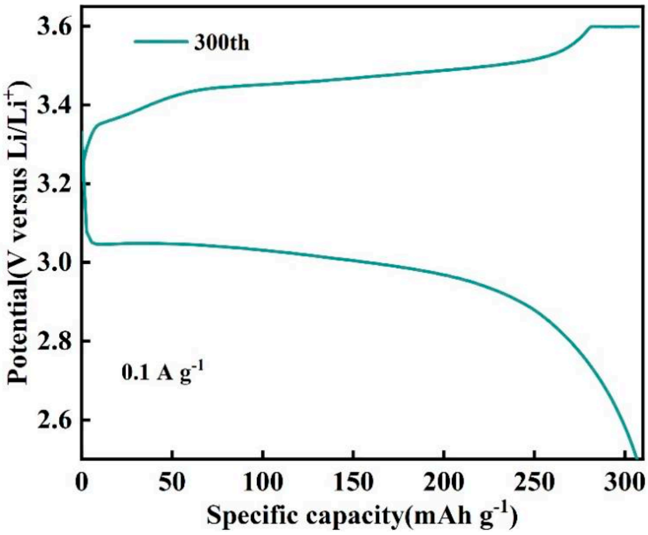

Figure S4. Charge/discharge profiles of full cells at 0.1 A g^{-1}.

Table S1. Comparisons of the synthetic method, morphology, cycle number, current density, and capacity between the Si@SiC@EG anode and other previously reported Si-based LIBs anodes.

Material	Method	Morphology	Current density (mA g^{-1})	Cycle number	Capacity (mAh g^{-1})
**Si@SiC@EG (this work)**	thermal decomposition	particles	100	160	1747
			1000	2000	872
**SiC@HGSs [6]**	surface graphitization	nanoshells	600	600	1345
			3000	1000	742
**SiO_2 [7]**	mechanical milling	particles	-	200	800
**SiO_x/C [8]**	CVD	particles	500	500	972
**SiC-Sb-C [9]**	mechanical milling	microspheres	2000	120	440
**SiO_x/C [10]**	sand milling	particles	325	500	645
**SiC/C [11]**	pyrolysis	nanofibers	100	250	254.5
**$SiN_{0.92}$ [12]**	pulsed laser deposition	thick films	0.02 C	100	700
**SiN_x@Si [13]**	vacuum CVD	nanocomposite	500	200	1400
**C@SiO_x [14]**	graphitization	nanospheres	5000	500	350
**NC@SiO_x [15]**	directly calcining	nanosheets	5000	1000	427.6

S6. References

1. Kresse, G.; Furthmüller, J., Efficient iterative schemes for ab initio total-energy calculations using a plane-wave basis set. *Phys. Rev. B* **1996,** 54, (16), 11169-11186.
2. Perdew, J. P.; Burke, K.; Ernzerhof, M., Generalized gradient approximation made simple. *Phys. Rev. Lett.* **1996,** 77, (18), 3865-3868.
3. Dudarev, S. L.; Botton, G. A.; Savrasov, S. Y.; Humphreys, C. J.; Sutton, A. P., Electron-energy-loss spectra and the structural stability of nickel oxide: an LSDA+U study. *Phys. Rev. B* **1998,** 57, (3), 1505-1509.
4. Henkelman, G.; Uberuaga, B. P.; Jónsson, H., A climbing image nudged elastic band method for finding saddle points and minimum energy paths. *J. Chem. Phys.* **2000,** 113, (22), 9901-9904.
5. He, T.; Feng, J.; Ru, J.; Feng, Y.; Lian, R.; Yang, J., Constructing heterointerface of metal atomic layer and amorphous anode material for high-capacity and fast lithium storage. *ACS Nano* **2018,** 13, 830-838.
6. Li, H.; Yu, H.; Zhang, X.; Guo, G.; Hu, J.; Dong, A.; Yang, D., Bowl-like 3C-SiC nanoshells encapsulated in hollow graphitic carbon spheres for high-rate lithium-ion batteries. *Chem. Mater.* **2016,** 28, (4), 1179-1186.
7. Chang, W.-S.; Park, C.-M.; Kim, J.-H.; Kim, Y.-U.; Jeong, G.; Sohn, H.-J., Quartz (SiO_2): a new energy storage anode material for Li-ion batteries. *Energy Environ. Sci.* **2012,** 5, (5), 6895-6899.
8. Liu, Z.; Zhao, Y.; He, R.; Luo, W.; Meng, J.; Yu, Q.; Zhao, D.; Zhou, L.; Mai, L., Yolk@shell SiO_x/C microspheres with semi-graphitic carbon coating on the exterior and interior surfaces for durable lithium storage. *Energy Storage Mater.* **2019,** 19, 299-305.
9. Chen, Z.; Cao, Y.; Qian, J.; Ai, X.; Yang, H., Antimony-coated SiC nanoparticles as stable and high-capacity anode materials for Li-ion batteries. *J. Phys. Chem. C* **2010,** 114, (35), 15196-15201.
10. Xu, Q.; Sun, J.-K.; Yin, Y.-X.; Guo, Y.-G., Facile synthesis of blocky SiO_x/C

with graphite-like structure for high-performance lithium-ion battery anodes. *Adv. Funct. Mater.* **2018,** 28, (8), 1705235.

11. Sun, X.; Shao, C.; Zhang, F.; Li, Y.; Wu, Q.-H.; Yang, Y., SiC nanofibers as long-life lithium-ion battery anode materials. *Front. Chem.* **2018,** 6, 166.
12. Suzuki, N.; Cervera, R. B.; Ohnishi, T.; Takada, K., Silicon nitride thin film electrode for lithium-ion batteries. *J. Power Sources* **2013,** 231, 186-189.
13. de Guzman, R. C.; Yang, J.; Ming-Cheng Cheng, M.; Salley, S. O.; Ng, K. Y. S., High capacity silicon nitride-based composite anodes for lithium ion batteries. *J. Mater. Chem. A* **2014,** 2, (35), 14577-14584.
14. Zhu, G.; Zhang, F.; Li, X.; Luo, W.; Li, L.; Zhang, H.; Wang, L.; Wang, Y.; Jiang, W.; Liu, H. K.; Dou, S. X.; Yang, J., Engineering the distribution of carbon in silicon oxide nanospheres at the atomic level for highly stable anodes. *Angew. Chem. Int. Ed.* **2019,** 58, (20), 6669-6673.
15. Guo, X.; Zhang, Y.-Z.; Zhang, F.; Li, Q.; Anjum, D. H.; Liang, H.; Liu, Y.; Liu, C.-s.; Alshareef, Husam N.; Pang, H., A novel strategy for the synthesis of highly stable ternary SiO_x composites for Li-ion-battery anodes. *J. Mater. Chem. A* **2019,** 7, (26), 15969-15974.
